# 

**Published:** 1898-01

**Authors:** 


### Southern Medical Record

A Monthly Journal of Medicine and Surgery.

Vol. XXVIII. ATLANTA, GA., JANUARY, 1898.

BERNARD WOLFF, M. D., EDITOR.

ASSOCIATE EDITORS:

. W. GRIGGS, M. D.
J. McFADDEN GASTON, M. D.
WILLIS F. WESTMORELAN

B. M. ZETTLER, BUSINESS MANAGER.

Entered at the Post-office at Atlanta, Ga., as Second-class Matter.

The Foote & Davies Co., Atlanta, Ga.

